# Leptin, its receptor and aromatase expression in deep infiltrating endometriosis

**DOI:** 10.1186/s13048-015-0180-0

**Published:** 2015-08-05

**Authors:** Helder F. Gonçalves, Carolina Zendron, Fernanda S. Cavalcante, Verônica Aiceles, Marco Aurélio P. Oliveira, Jorge Henrique M. Manaia, Márcio A. Babinski, Cristiane F. Ramos

**Affiliations:** Laboratory of Morphometry, Metabolism and Cardiovascular Disease, Biomedical Center, Institute of Biology, Department of Anatomy, State University of Rio de Janeiro, Rio de Janeiro, Brazil; Department of Gynecology, State University of Rio de Janeiro, Rio de Janeiro, Brazil; Departament of Morfology, Biomedical Institute, Fluminense Federal University, Niterói, Rio de Janeiro Brazil

**Keywords:** Endometrial implants, Endometriosis, Peritoneal fluid, Leptin, Leptin receptor, Aromatase

## Abstract

**Background:**

The aim of this study was to evaluate the leptin levels in the serum and peritoneal fluid (PF) and the protein expression in three different peritoneal ectopic implants in patients who underwent surgery for deep infiltrating endometriosis.

**Methods:**

All patients had been treated at the Department of Gynecology of the Pedro Ernesto University Hospital, Rio de Janeiro. The study group consisted of 15 patients who underwent surgery for adnexal masses and infertility, while the control group consisted of ten women who underwent surgery for tubal ligation. Peritoneal fluid and samples tissues were collected during surgery. Serum samples were obtained before anesthesia. In this study, the leptin levels in the serum and peritoneal fluid (PF) were evaluated by ELISA. The protein expression of leptin and its receptors (ObR) and aromatase enzyme were evaluated by Western blot analysis of the intestine, uterosacral ligament and vaginal septum in the ectopic implants. The *t*-test and one-way ANOVA with Holm-Sìdak post-test were used, and p < 0.05 was considered to be statistically significant.

**Results:**

Compared to the controls, the serum leptin levels (control = 14.7 ng/mL ± 2.63, endometriosis = 19.2 ng/mL ± 1.84, p < 0.0001) were increased, while in PF, there was no difference (control = 6.68 ng/mL ± 0.43, endometriosis = 7.71 ng/mL ± 0.59, p = 0.18). Comparing women with and without ovarian implants, the leptin levels in both the serum and PF were significantly higher in women without ovarian implants (serum: with ovarian implant = 15.85 ± 1.99; without ovarian implant = 23.14 ± 2.60; ng/mL, p = 0.04; PF: with ovarian implant = 4.28 ± 1.30; without ovarian implant = 11.18 ± 2.98;ng/mL, p = 0.048). The leptin, ObR and aromatase protein expression levels were increased in lesions in the vaginal septum and were decreased in the intestine lesions.

**Conclusion:**

This study reports several interesting associations between the leptin levels in serum, peritoneal fluid, and tissue samples and the localization of the ectopic endometrium. Although this study does not provide a clear picture of the role of leptin in the development and progression of peritoneal implants, it contributed new data that might be useful to elucidating the enigma that is the role of leptin in endometriosis disease.

## Background

Endometriosis is a chronic, estrogen-dependent inflammatory disease characterized by implantation and growth of tissue outside of the uterus in lesions of varying sizes and appearance containing endometrial glands and stroma associated, or not, with infertility, dysmenorrhea, and pelvic pain [1–3]. This disease represents a public health issue affecting 10–15 % of women of reproductive age [4]. Studies have demonstrated that endometriosis is associated with the increased secretion of pro-inflammatory cytokines and growth as well as angiogenic factors [5].

Leptin, the 16 kDa product of the Obese (Ob) gene, is predominantly produced in adipose tissue [6]. This hormone is known for its role in food intake, basal metabolism, and reproductive function [7]. Leptin is now recognized to have immune-regulatory, pro-inflammatory, and neoangiogenesis functions, and it is thought to have a role in the pathogenesis of endometriosis [5, 8–12].

Studies evaluating the serum and peritoneal fluid (PF) levels of leptin in patients with endometriosis have reported conflicting results. Both high [5, 13–18] or unchanged levels [15, 19–22] have been reported. Moreover, the possibility of an association between the PF leptin levels and severity of endometriosis is also controversial; some studies have suggested a negative correlation [5, 14, 16], while others have shown a positive correlation with more severe forms of peritoneal endometriosis [11, 13, 15, 22].

Interestingly, only a few studies thus far have evaluated the leptin receptor gene and/or protein expression in the endometrial tissue of women with endometriosis [12, 23, 24], but none have evaluated the leptin expression in ectopic lesions.

Another interesting finding about leptin and endometriosis is that patients with peritoneal implants have higher PF leptin concentrations than women in whom no implant was observed, suggesting that leptin may play a role in the development of peritoneal endometriosis and that different biochemical phenomena might be involved in the pathogenesis of the ovarian form of the disease [14].

In view of the potential role of leptin in peritoneal endometriosis, we evaluated the leptin levels in the serum and peritoneal fluid and the protein expression in three different peritoneal ectopic implants in patients who underwent surgery for severe endometriosis.

## Materials and methods

### Patient enrollment

The study group consisted of patients who underwent laparotomy or laparoscopy for adnexal masses and infertility. The inclusion criteria for this group were stage IV endometriosis, at least one year of primary infertility, pain, and regular cycles before starting hormonal treatment. Several peritoneal endometriotic implants were observed in all 15 patients included in this group, and an ovarian implant was also found in eight of the patients. The control group consisted of ten women with proven fertility from the family-planning program of the same hospital who were undergoing mini-laparotomy or laparoscopy for tubal ligation and who lacked surgical evidence of endometriosis or any ovarian pathology. All patients in the control group had a normal pelvic cavity.

The surgeries were performed between February 8, 2013 and July 31, 2013, at the Department of Gynecology of the Pedro Ernesto University Hospital, Rio de Janeiro.

All patients were of reproductive age and were receiving hormonal therapy for the clinical treatment of pain associated with endometriosis or for contraception (control group). All enrolled patients had a body mass index (BMI) of 20.4 to 25.4 kg/m^2^.

The exclusion criteria were clinical and/or echographic indications of diabetes, polycystic ovarian disease and surgical evidence of any other genital pathology.

This study was approved by the local ethics committee (C.E.P.: 195.160, Pedro Ernesto University Hospital, Rio de Janeiro, Brazil). Before the procedures, all patients signed informed consent.

### Tissue specimens

Serum samples were obtained from all patients before anesthesia. The PF samples were aspirated from the Douglas pouch at the beginning of surgery; however, some patients had a very low level of fluid, which made the aspiration method impossible. In case of hemorrhage after the insertion of trocars, the samples were not collected to avoid blood contamination. As a result, only 9 patients from the peritoneal endometriosis group and 8 patients from the control group had their PF samples analyzed. The same surgeon who performed cystectomy removed the ovarian endometrioma. Peritoneal biopsies of endometriosis were confirmed by histology in all patients with suspected implants at laparoscopy and were classified according to the classification of the American Society of Reproductive Medicine [25].

### Western blotting

Approximately 500 mg of tissue was homogenized in 500 μL of lysis buffer containing 1% NP-40 (Amresco, Ohio, USA) and a protease-inhibitor mix (Sigma-Aldrich, USA); then, the tissue was centrifuged at 9,700 rpm at 4 °C. The protein concentration was measured by fluorometry (Qubit 2.0, Life Technologies Corporation, CA, USA), and 20 μg aliquots were applied to 8% SDS-polyacrylamide gel and submitted to vertical electrophoresis; they were then transferred to nitrocellulose membranes in a semi-dry transfer apparatus. The membranes were subsequently incubated with antibodies (Santa Cruz, CA, USA) to leptin (1:500), ObR (1:200) and aromatase (1:250). The expression of the proteins under study was normalized against the expression of β-actin. The bands were visualized by chemiluminescence (ECL, Amersham Biosciences, Piscataway, NJ, USA) and documented on the ChemiDoc MP System, Bio-Rad (Life Science Research, USA). All bands were quantified using Image J software 1.42q, USA.

### Leptin and estradiol levels

The concentration (ng/mL) of leptin in the serum and PF was determined by ELISA (Millipore Corporation Billerica, MA, USA). The concentrations (pg/mL) of estradiol in the serum and peritoneal fluid were measured using chemiluminescence (Kit Cobas, Roche Diagnostics, USA) at the hormone laboratory of the Pedro Ernesto University Hospital, Rio de Janeiro. The sensitivities of the assays were 0.195 ng/mL for leptin and 5.00 pg/mL for estradiol.

### Statistical analyses

Pearson’s correlation coefficient was used to evaluate the relationship between the leptin levels and PF estradiol levels, aromatase expression and estradiol levels. The *t*-test was used to evaluate the differences between two groups. One-way ANOVA with the Holm-Sìdak post-test was used when 3 groups were compared. All results are reported as the mean ± standard error of the mean (SEM), and p-values < 0.05 were considered to be statistically significant. The calculations were performed with GraphPad Prism (version 6.0, GraphPad Software, CA, USA).

## Results

### Age, BMI and ectopic implant number

The age, BMI and ectopic implant number for each subject are shown at Table 1. All patients were classified as having stage IV endometriosis. One inclusion criterion was the use of hormone therapy; 87 % of patients in the study group and 60 % of patients in the control group were using a combined oral contraceptive (progesterone and estrogen), and the remaining patients in both groups were using isolated progestin therapy.

**Table 1:** Baseline characteristics of the patients and the ectopic implant numbers

	Age	BMI (kg/m^2^)	Ectopic implant numbers
Control group (n = 10)	33.10 ± 2.32	24.37 ± 0.81	-
Endometriosis group (n = 15)	32.87 ± 1.16	22.51 ± 1.60	2.6 ± 0.33

Values are given as the mean ± standard error of the mean (SEM). The number in parentheses corresponds to the number of samples. There was no significant difference between the two groups

### Hormone levels

**Fig. 1:**
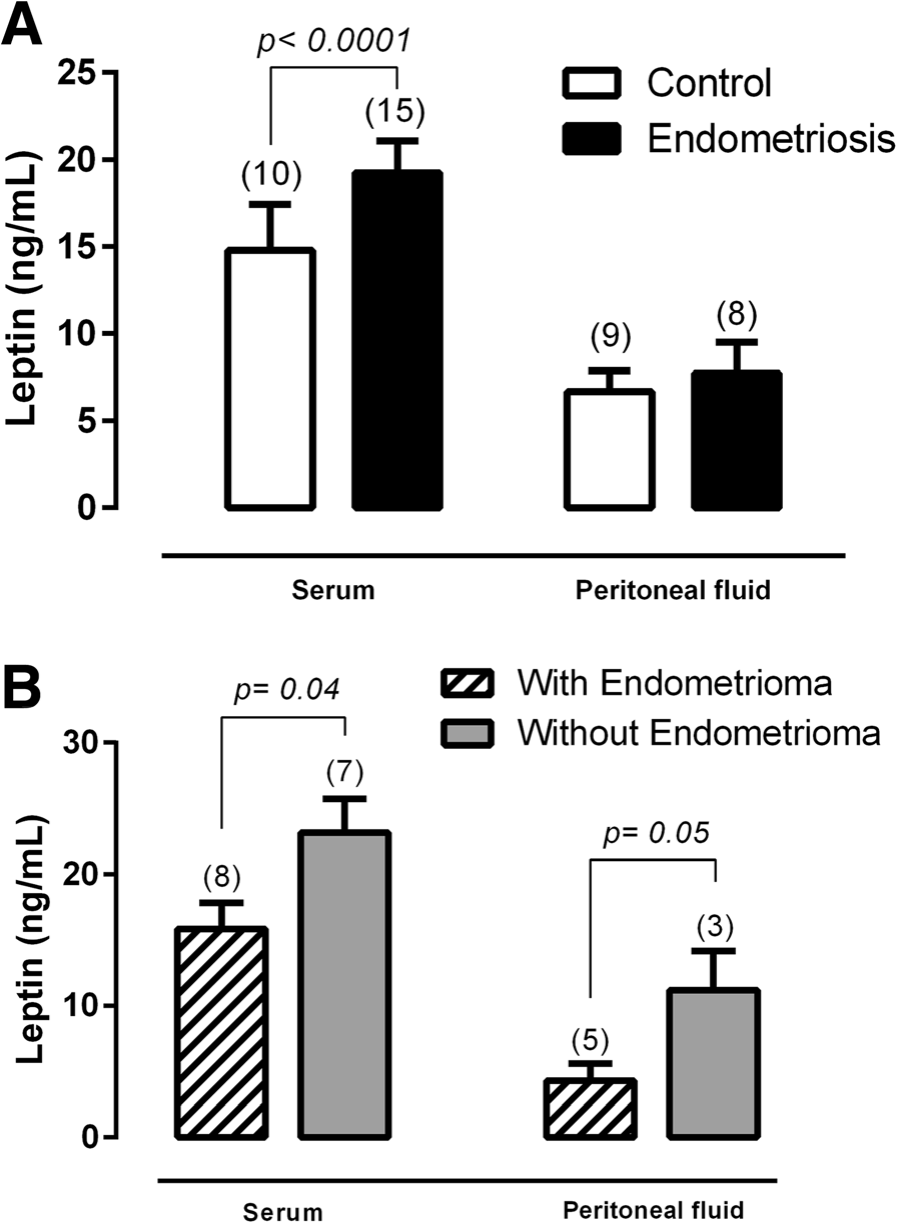
The serum and peritoneal fluid leptin levels in control (white bar) and peritoneal endometriosis (black bar) women (**a**). The serum and peritoneal fluid leptin levels in peritoneal endometriosis patients with and without ovarian implants (**b**). Values are given as the mean ± standard error of the mean (SEM). The number in parentheses corresponds to the number of samples

Considering all fifteen patients, the peritoneal endometriosis group presented with significantly high serum leptin levels compared to the control group (control = 14.7 ± 2.63, endometriosis = 19.25 ± 1.84, ng/mL, p < 0.0001). In contrast, the leptin levels in the PF were not different (control = 6.68 ± 0.43, endometriosis = 7.71 ± 0.59, ng/mL, p = 0.18) (Fig. 1a). However, comparing the women with and without ovarian implant, both the serum (with ovarian implant = 15.85 ± 1.99; without ovarian implant = 23.14 ± 2.60; ng/mL, p = 0.04) and PF (with ovarian implant = 4.28 ± 1.30; without ovarian implant = 11.18 ± 2.98;ng/mL, p = 0.05) leptin levels were significantly higher in the women who did not have an ovarian implant (Fig. 1b).

**Fig. 2:**
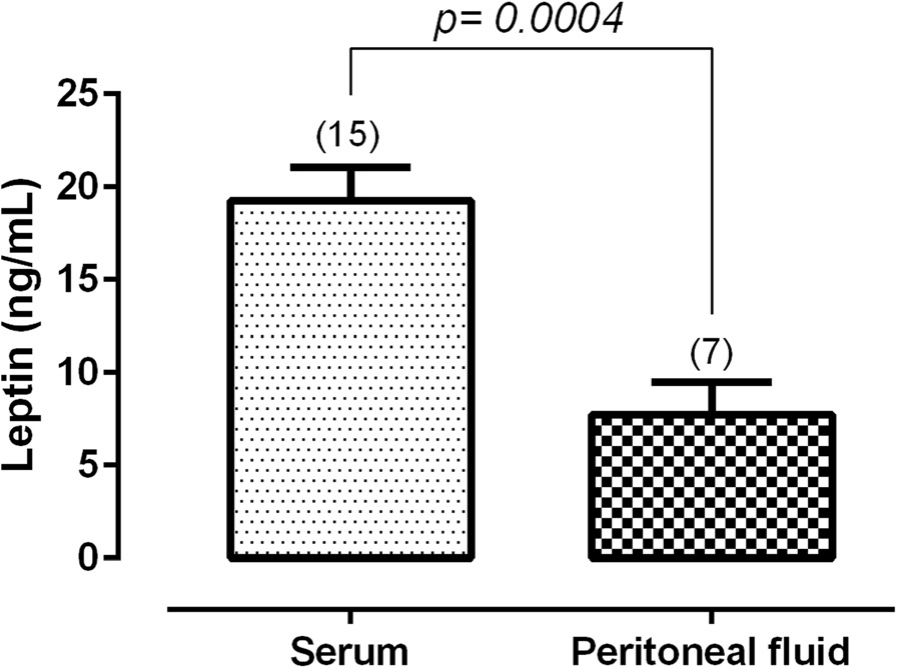
The leptin levels in the serum and peritoneal fluid of patients in the peritoneal endometriosis group. Values are given as the mean ± standard error of the mean (SEM). The number in parentheses corresponds to the number of samples

**Fig. 3:**
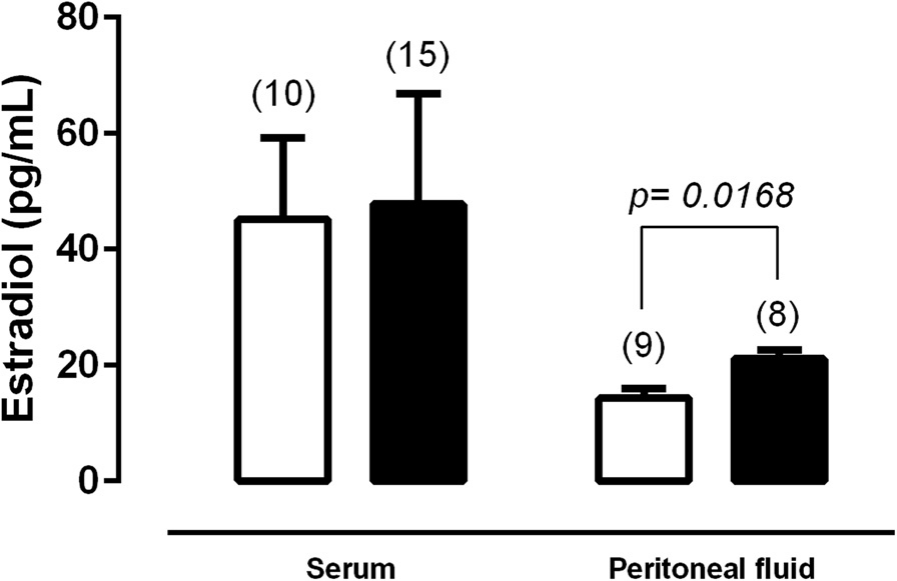
The serum and peritoneal fluid estradiol levels in control (white bar) and peritoneal endometriosis (black bar) groups. Values are given as the mean ± standard error of the mean (SEM). The number in parentheses corresponds to the number of samples

The leptin levels in the serum and PF of patients in the peritoneal endometriosis group are presented in Fig. 2. The leptin levels in the PF were significantly lower than those in the serum (serum = 19.25 ± 1.84; PF = 7.72 ± 1.78; ng/mL, p = 0.0004). Figure 3 shows there is no difference in the estradiol serum levels between the endometriosis and control groups (control = 45.17 ± 14.11; endometriosis = 47.83 ± 18.92; pg/mL, p = 0.9192). However, in the control group, the PF estradiol levels were lower than in the endometriosis group (control = 14.30 ± 1.71; endometriosis = 21.15 ± 1.56; pg/mL, p = 0.0168).

### Protein expression

**Fig. 4:**
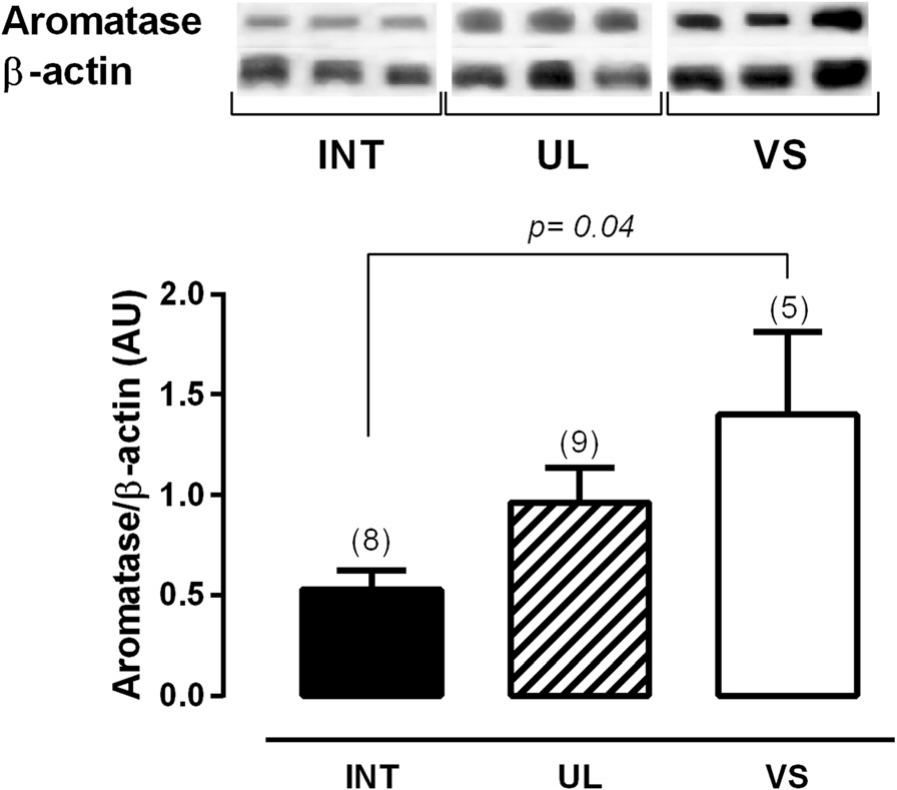
Twenty micrograms of protein were resolved on SDSPAGE, which was followed by immunoblot analysis with specific antibodies against aromatase in intestine lesions (INT), uterosacral ligament lesions (UL) and vaginal septum lesions (VS) in peritoneal endometriosis patients. Representative bands of results are provided above the graphs. Β-actin was used as an internal control. Values are given as the mean ± standard error of the mean (SEM). The number in parentheses corresponds to the number of samples

**Fig. 5:**
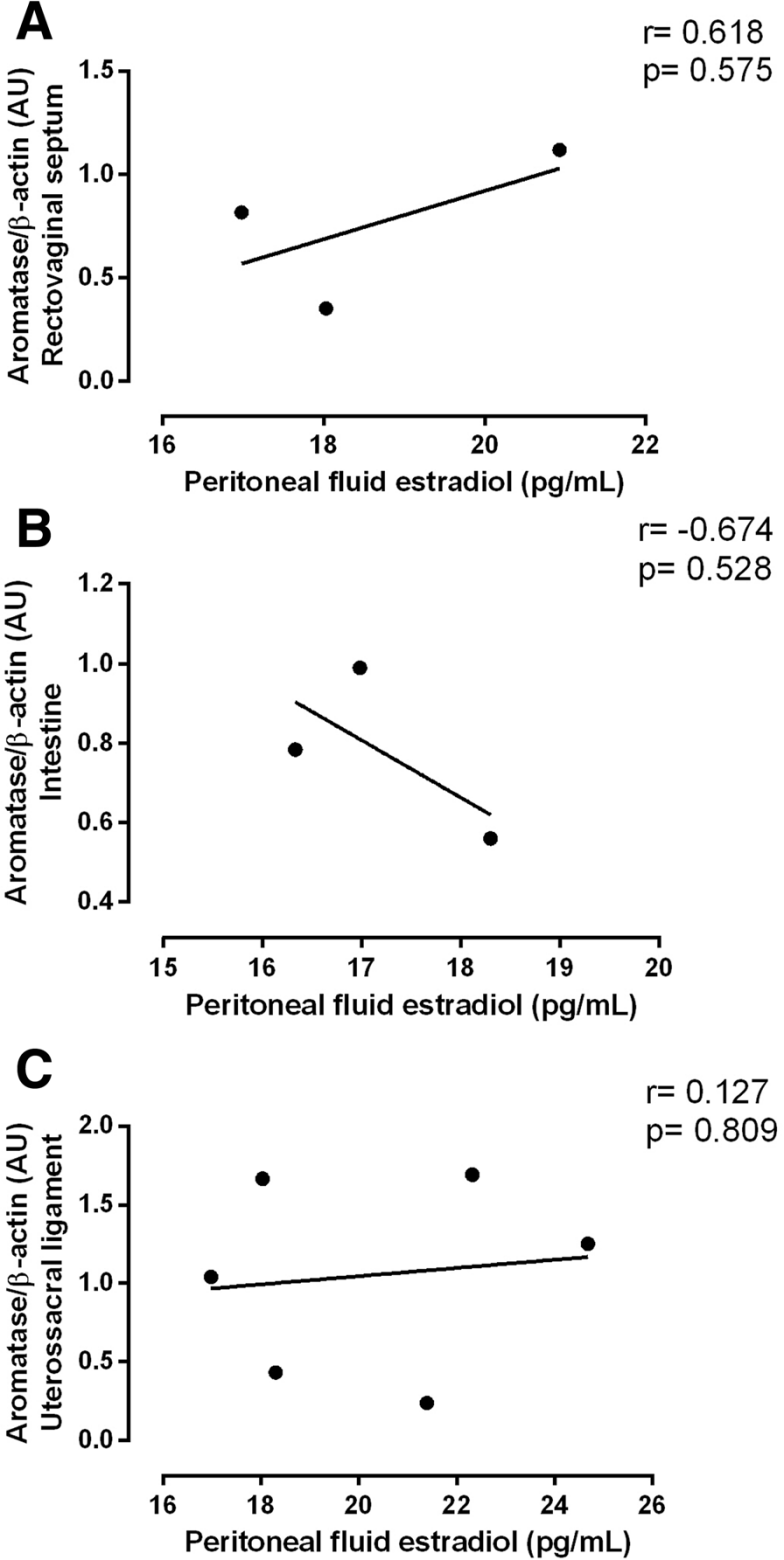
Pearson’s correlation between the protein aromatase expression and peritoneal fluid estradiol levels in the vaginal septum (**a**), intestine (**b**) and uterosacral ligament (**c**)

**Fig. 6:**
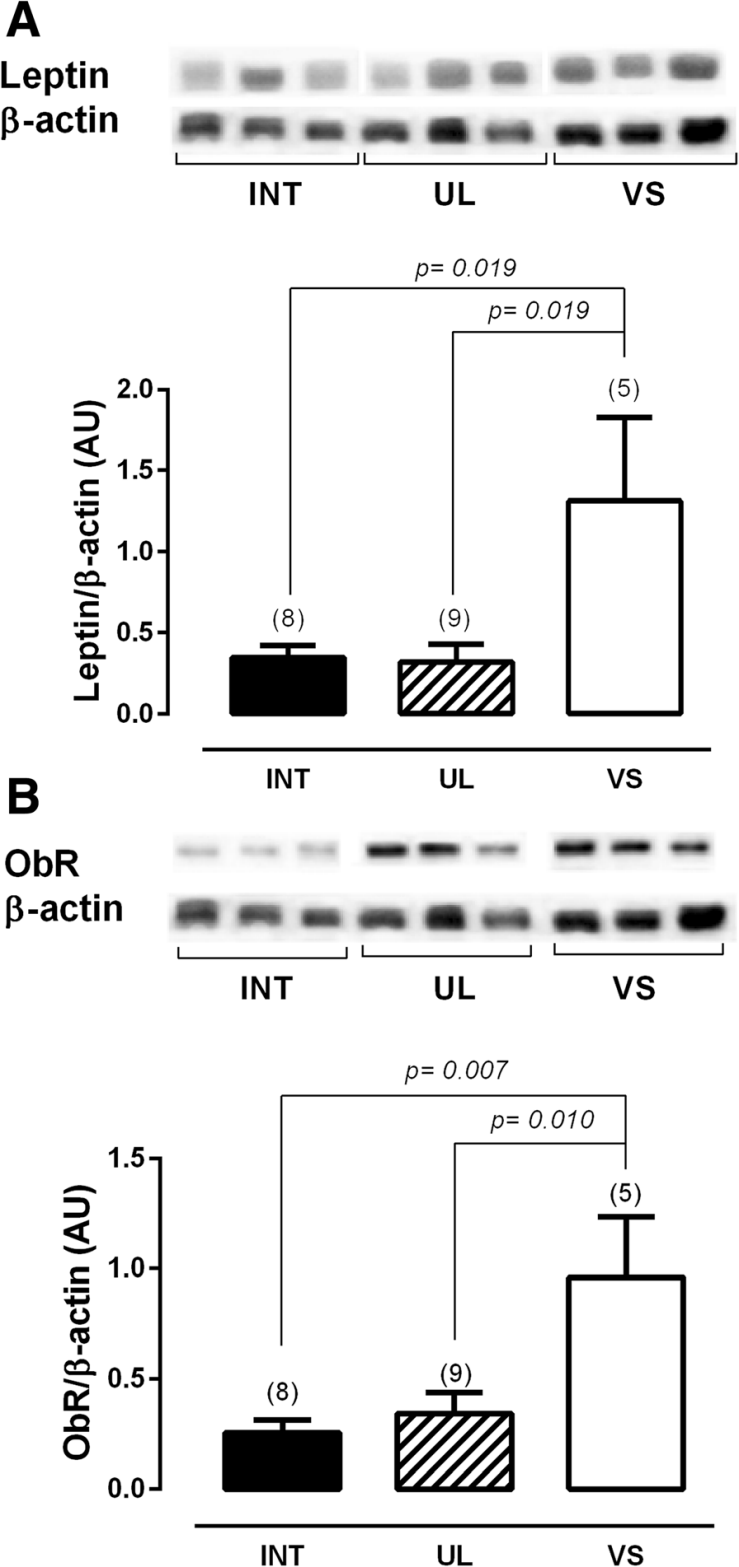
Twenty micrograms of protein were resolved on SDSPAGE, which was followed by immunoblot analysis with specific antibodies against leptin (**a**) and ObR (**b**) in intestine lesions (INT), uterosacral ligament lesions (UL) and vaginal septum lesions (VS) in peritoneal endometriosis patients. Representative bands of the results are shown above the graphs. β actin was used as an internal control. Values are given as the mean ± standard error of the mean (SEM). The number in parentheses corresponds to the number of samples

The protein expression of aromatase shows that the implants found in the vaginal septum have the highest expression level, while the intestine implants have the low levels of this enzyme. The implants found in the uterosacral ligaments had intermediate values (Fig. 4). Additionally, this expression does not seem to correlate with the estradiol levels in the PF (Fig. 5). The protein expression of leptin and its receptor, ObR, had the same pattern of higher expression in the vaginal septum lesions and lower expression in the intestine lesions (Fig. 6).

## Discussion

Leptin, a hormone that is mainly produced by adipocytes [6], is also expressed in endometrium and has been implicated in endometriosis [26]. The interest in the understanding of the association between leptin and endometriosis has been increasing. An interesting finding about leptin and endometriosis is that patients with peritoneal implants at all stages of endometriosis show higher PF leptin concentrations than women in whom no implant was observed, suggesting that leptin may play a role in the development of peritoneal endometriosis [14].

However, the studies found in the literature on this topic are controversial, which could be related with the variability in the control groups. Women usually included in this group undergo surgery for infertility without a known cause [5, 14, 21] or tubal ligation [5, 13, 14, 20], miomas [20], or other pathologies that are different from endometriosis [13, 14, 26]. In an attempt to reduce the bias of this control, the endometriosis group in this study consisted of women with stage IV endometriosis. For the control group, we recruited subjects from among women who were already scheduled to undergo surgery for tubal ligation. In this line of thinking, all women of both groups were under hormonal therapy, reducing the hormonal variations and influences of the menstrual cycle, which was confirmed by the similar estradiol serum values between the groups. Another inclusion criterion was BMI. Only women with BMI values between 20.4 to 25.4 kg/m^2^ were included in this study because endometriosis is inversely associated with BMI [27, 28].

Studies evaluating the serum and peritoneal fluid levels of leptin in patients with endometriosis report conflicting results of both high levels [5, 13–18] or unchanged levels [15, 19–22]. No studies in the literature have evaluated stage IV patients alone. Again, we believe that the variability among the studies in relation to patient criteria is responsible for the discrepancy in the results. In addition to the criteria already discussed for the bias in the control group, the studies reported great variability in the endometriosis group in relation to BMI, use or not of hormonal therapy and stage of disease. All of these variables are responsible for the controversy in the literature, which makes the role of leptin in the pathology of endometriosis an enigma.

Recently we have published a study on endometriosis stage IV patients [29] in which we found that that leptin levels in both the serum and PF were changed. In addition, in the cystic fluid of endometrioma, the leptin levels were very high compared to the serum and PF. In this study, we showed that endometriosis patients presented with high levels of serum leptin, while the peritoneal fluid levels were unchanged compared to the control ones. We could attribute the difference in the leptin serum levels to the high number of patients enrolled in the present study.

Analyzing leptin in women with peritoneal endometriosis with and without ovarian implants, we found that in peritoneal endometriosis without ovarian implants, the leptin serum and peritoneal fluid levels were higher than in women with ovary implants. Together with data from literature [13], these data suggest that the presence of peritoneal disease, and not of an ovarian implant, is the factor that influences the concentration of leptin in the serum and PF in endometriosis. This result and the fact that the cystic fluid of endometrioma has high levels of leptin compared to serum and PF [29] suggest that the leptin produced by the ovary implant could be sequestered to the endometrial cyst instead of going to the serum or peritoneal fluid.

The leptin regulation by steroid is another controversial point. Data from animal models suggest that gonadal steroids modulate leptin expression [30–32]. The leptin concentrations are higher among women than among men, which is explained by the positive regulation of estrogen on leptin secretion, while androgen negatively regulates these levels [33–36]. In addition, the increased fat depots in women could also explain this gender difference [37]. The leptin levels in premenopausal women are higher than those in postmenopausal women [38]. Additionally, it is known that leptin can either vary during the menstrual cycle, higher at the luteal phase [31, 39] or not, even in women under hormonal therapy [5, 40, 41]. The endometriosis group showed no difference in the estradiol serum levels, while the peritoneal fluid is lower than in the control group. Based on these results and the fact that estrogen positively regulates leptin, the endometriosis group should have normal serum and low PF leptin levels. Therefore, we could assume that this regulation is missed in these endometriosis patients. More studies are in development in our lab to establish a possible relationship between leptin expression and the number or site of focus.

It is known that ectopic endometriotic tissues have estrogen-producing ability [31, 42, 43]. In attempt to correlate the estradiol production by each ectopic implant with the peritoneal fluid estradiol level, the aromatase expression was evaluated. Although there was a non-significant correlation between peritoneal fluid estradiol and a local ectopic implant aromatase expression (most likely by the small number of samples), the results suggest a positive correlation in the vaginal septum implant and a negative correlation in the intestine implant. However, in spite of the fact that aromatase enzyme seems to be expressed differently in each ectopic endometriotic implant, it is not sufficient to explain why its expression is lower compared to the control women. Further analysis is necessary to evaluate whether the healthy tissue that surrounds the implant can have a regulatory role on disease progression.

In relation to the leptin expression, the results were very similar with those for aromatase. Both leptin and its receptor, ObR, were higher in the vaginal septum implant compared to the intestine implant. The implant found in the uterosacral ligament expresses medium values of both proteins. A recent paper reported that the peritoneal implants express high leptin and ObR than eutopic endometrium [44]. We did not find any paper that compared the expression of aromatase, leptin or ObR among the different peritoneal ectopic implants; however, because leptin has angiogenic and immunologic proprieties [45, 46], the high expression of both leptin and its receptor could be related to the migration of the endometrium to its implant site.

## Conclusion

This study reports some interesting associations between the leptin levels in the serum, peritoneal fluid, tissue samples and the localization of the ectopic endometrium. Although this study does not give a clear picture of the role of leptin in the development and progression of peritoneal implants, it contributes new data that might be useful to elucidating the enigma that is the role of leptin in endometriosis disease.

## Abbreviations

PF: Peritoneal fluid
ObR: Leptin receptor
Ob: Obese gene
BMI: Body mass index
SEM: Standard error of the mean
